# Common knowledge promotes risk pooling in an experimental economic game

**DOI:** 10.1371/journal.pone.0220682

**Published:** 2019-08-15

**Authors:** Lee Cronk, Athena Aktipis, Steven Gazzillo, Dave White, Amber Wutich, Barry Sopher

**Affiliations:** 1 Department of Anthropology, Rutgers University, New Brunswick, New Jersey, United States of America; 2 Department of Psychology, Arizona State University, Tempe, Arizona, United States of America; 3 Department of Economics, Rutgers University, New Brunswick, New Jersey, United States of America; 4 Decision Center for a Desert City, Arizona State University, Tempe, Arizona, United States of America; 5 School of Human Evolution and Social Change, Arizona State University, Tempe, Arizona, United States of America; Universidad Loyola Andalucia Cordoba, SPAIN

## Abstract

Risk management is a problem humans have faced throughout history and across societies. One way to manage risk is to transfer it to other parties through formal and informal insurance systems. One informal method of self-insurance is limited risk pooling, where individuals can ask for help only when in need. Models suggest that need-based transfer systems may require coordination and common knowledge to be effective. To explore the impact of common knowledge on social coordination and risk pooling in volatile environments, we designed and ran a Risk Pooling Game. We compared participants who played the game with no advance priming or framing to participants who read one of two texts describing real-world systems of risk pooling. Players in the primed games engaged in more repetitive asking and repetitive giving than those in the control games. Players in the primed games also gave more in response to requests and were more likely to respond positively to requests than players in the control games. In addition, players in the primed games were more tolerant of wide differences between what the two players gave and received. These results suggest that the priming texts led players to pay less attention to debt and repayment and more attention to the survival of the other player, and thus to more risk pooling. These results are consistent with findings from fieldwork in small-scale societies that suggest that humans use need-based transfer systems to pool risk when environmental volatility leads to needs with unpredictable timing. Models suggest that the need-based transfer strategy observed in this experiment can outperform debt-based strategies. The results of the present study suggest that the suite of behaviors associated with need-based transfers is an easily triggered part of the human behavioral repertoire.

## Introduction

Human responses to uncertainty fall into four main risk management strategies [1]. *Risk retention* consists of accepting risk and absorbing any resulting losses. This includes the accumulation and storage of resources, whether by individuals or groups, and institutional self-insurance. Risk retention may be most appropriate when losses occur frequently but are not very severe [2]. *Risk avoidance* involves reducing dependence on high variability outcomes. For example, pastoralists may avoid risk by reducing their reliance upon herds, which are subject to occasional catastrophic losses, and practicing other, more reliable forms of subsistence, such as farming (e.g.,[3]). *Risk reduction* refers to efforts to lower the probability of losses or to reduce the size of losses. For example, pastoralists may vaccinate their livestock to protect them from diseases and build fences to protect them from predators and thieves. *Risk transfer* is the exchange of risk from one individual or group to another. In market economies, risk is often transferred through the purchase of commercial insurance policies. Commercial insurance was made possible by the existence of actuarial databases. Such databases enable insurance companies to set premiums at levels that will allow them to cover their costs. Despite uncertainty about the likelihood that any individual will suffer a loss, insurers can be relatively confident about the likelihood that a category of people will suffer one [4].

In the absence of commercial insurance, risk transfer takes place through social networks [5]. The resulting risk pooling (also known as risk sharing) arrangements arise when two or more parties agree to take on some portion of one another’s risk. Participants in such arrangements are essentially exchanging the small likelihood of a catastrophic loss for the high likelihood of small, manageable losses. This is analogous to the exchange made in commercial insurance of a certain but small and manageable loss in the form of a premium in exchange for coverage in the unlikely event of a large, catastrophic loss. Although commercial insurance has many advantages, risk pooling arrangements also have some notable strengths. For example, insurance policies are very specific, insuring against a single risk or a narrowly defined set of risks and cover a single asset or a narrowly defined set of assets. In contrast, grassroots risk-pooling arrangements can be much more flexible, with a single relationship covering a wide range of assets. Furthermore, in risk-pooling arrangements, the risks covered do not always need to be fully specified in advance [6].

There are long traditions of empirical work on risk pooling arrangements in both economics (e.g., [5, 7–12]) and anthropology (e.g., [13–21]). Our own work on this topic, which we conduct under the auspices of the Human Generosity Project (http://humangenerosity.org), began with a system of risk pooling among Maasai pastoralists in East Africa that they refer to as *osotua*. Although osotua’s original and literal meaning is a human umbilical cord, Maasai use it metaphorically to refer to the dyadic relationships that are at the heart of their system of risk pooling [6,22–26].

Maasai parents begin to instill osotua values in their children during childhood by encouraging them to form friendships known as *isirito* (singular: *esirit*), within which children share food and exchange small gifts. Esirit-type friendships may lead to transfers in adulthood of much more valuable gifts, such as individual livestock. The overall process is similar to courtship, with prospective osotua partners getting to know each other and giving small gifts over a period of years. When a degree of trust has been established, the individuals involved may agree to be osotua partners. Most people establish multiple osotua partnerships. People often seek osotua relationships with people whose risk profiles are complementary to their own. For example, people living in the drought-prone lowlands seek osotua partners in the wetter highlands, and vice versa. Similar patterns in the formation of stock friendships (i.e., special friendships based on rules governing livestock transfers between parties) have also been observed among pastoralists elsewhere in East Africa (e.g., [27–33]).

Osotua transfers are initiated by a request from one partner to another. Such requests must arise only from genuine need and must be limited to the amount needed. Osotua requests are expected to be granted so long as the donor can afford to give. Gifts given in response to such requests should be given freely (*pesho*) and from the heart (*ol-tau*) but, like the requests, they are limited to what is needed [34]. Although gifts of livestock are the most common way that osotua partners help each other, virtually any good or service may serve as an osotua gift. Once an osotua relationship is established, it is, in principle at least, eternal. Even if the individuals who established the relationship die, it is supposed to be carried on by their children [35]. Osotua also does not follow a schedule and will not diminish even if much time passes between transfers. Although osotua relationships do involve reciprocal obligations to help if asked to do so, osotua transfers are not necessarily reciprocal or even roughly equal over extended periods of time. If one partner is consistently needier than the other, then the flow of goods and services in that relationship may be mostly or entirely one-way. Osotua relationships are imbued with a sense of respect (*enkanyit*), restraint, and responsibility in a way that non-osotua economic relationships (e.g., those based on debt) are not. One interviewee put it this way: “*Keiroshi*”: “It is heavy.”

The rules of osotua do not apply to all gift-giving among Maasai. Some gift-giving results instead in debt (*esile*). Osotua and esile are quite different. While osotua partners have an obligation to help each other in time of need, providing help to an osotua partner does not create debt [34,35]. In fact, osotua gifts are not payments at all, and so cannot and do not repay debt. In keeping with this logic, Maasai see it as inappropriate to use the verb “to pay” (*alak*) when referring to them. In contrast, when an animal is given under the rules of esile, repayment is expected in the form of an animal at least as valuable as the one given. Maasai refer to the repayment as *elaata*, which means to set free or untie a knot [34]. If a debtor fails to repay, his creditor may forgive the debt but then refer to him henceforth as *Pasile*: “One whose debt I have forgiven.” This type of construction, in which the prefix “pa” is used to indicate what a person has given or received, is more commonly used in a positive way. For example, a man refers to his son-in-law as “*Pakiteng*,” meaning “cow giver” (*pa + enkiteng*) in recognition of the bridewealth he paid. Thus, the use of the term “Pasile” serves as a mild rebuke to someone who has failed to repay a debt [6].

Maasai call upon their osotua partners for help or ask for a loan under the terms of esile under different circumstances. Requests to osotua partners may be made only when the requester is in serious need, e.g., when he has lost livestock to disease. Requests for loans under the rules of esile, in contrast, are typically made when a person is not in serious need in general but rather when he needs something very specific. For example, some ceremonies require the use of animals of particular colors, so a person who has no such animal in his herd might ask for a loan of such an animal, which he can then repay with an animal of equal or greater value but of a different color.

To examine the dynamics of risk pooling by pastoralists in volatile environments, members of the Human Generosity Project have developed a series of agent-based models [24–26] (see also [36]). These models have shown that agents following transfer rules based on the osotua system survive longer than agents following other transfer rules. The most recent of these models [25] was specifically designed to compare agents following transfer rules based on the osotua system with agents following rules based on the esile system. Agents in the model used one of three different decision-making rules for livestock transfers to one another: (1) no transfers at all, (2) transfers following the rules of osotua relationships (henceforth “need-based transfers”), and (3) transfers following the rules of esile (henceforth “debt-based transfers”). This model showed that, when environmental conditions are volatile, need-based transfers lead to more risk pooling and higher survivorship than either no transfers or debt-based transfers.

One particularly interesting insight that emerged from this research was that the choice between need-based transfers and debt-based transfers is a Stag Hunt Game (Table 1) [37,38]. In such a game, both need-based transfers and debt-based transfers are coordination points, with both players surviving longest if they both choose a need-based transfer strategy. Debt-based transfers is also a coordination point, but with a lower rate of survival. When one player uses one strategy and the other a different strategy, the result is instability, a lack of coordination, and a failure to achieve the desired outcome where both parties receive the highest payoff.

**Table 1:** Risk pooling norms as a Stag Hunt Game. In a computer simulation (Aktipis et al. 2016), need-based vs. debt-based strategies correspond to a Stag Hunt Game, with need-based transfers being dominant to debt-based transfers. Payoffs in this game theoretic matrix are given in percent of agents surviving at 50 years. Both players get the highest payoff if they both choose need-based transfers.

		Player 2	
		Need-based	Debt-based
Player 1	Need-based	30, 30	23, 29.5
	Debt-based	29.5, 23	25, 25

Thus, in the computer simulation the benefits from adopting a need-based transfer strategy come only to pairs that effectively coordinate. This and other coordination problems are solved when people have a shared understanding regarding how the problem may be solved [39,40]. For example, Chaudhuri et al. [41] ran an extended Stag Hunt-type game called the Minimum Effort Game [42] in a laboratory setting with different knowledge conditions. When participants had no common knowledge about how best to play the game, coordination quickly deteriorated, but when advice about how best to play the game was read aloud by the experimenter high rates of coordination were achieved and maintained over time. Similarly, Thomas et al. [43] ran a more traditional Stag Hunt game on Amazon Mechanical Turk. Once again, high rates of coordination were achieved when the experimenters announced to both players simultaneously that this was the time to coordinate on the more lucrative of two possible coordination points.

This leads to some interesting questions. How might common knowledge about the advantages of need-based transfers in volatile environments arise? How can individuals coordinate their behaviors to take advantage of the benefits of need-based transfer systems while avoiding the costs of engaging in need-based transfers when account-keeping is the norm? One possibility is that people are particularly sensitive to information about the coordination of social behavior because it is only by being sensitive to such information that people can reap the benefits of social coordination [44]. If so, then people may be more likely to engage in transfers based on need when provided with information about need-based transfer systems such as the osotua system of the Maasai. To better understand how such information shapes need-based transfer behavior, we designed an experimental economic game.

## Experimental procedures, design, and sample

This research was approved by Rutgers University's Institutional Review Board. Participants provided written informed consent. Our Risk Pooling Game is loosely based on a two-person agent-based model of osotua [24,25]. Players interact with one another, in anonymous pairings, via a computer interface. Each player has resources that can grow and shrink over time. Each player plays the game first during a seven-period practice session and then during four rounds consisting of twenty periods each. To earn money, players can harvest resources period by period. To remain in the game, players must maintain their resources above a specified threshold level. If a player’s resources fall below that threshold for three consecutive periods, then they are out of the game, unable to harvest and unable to request resources or respond to requests for resources. During game play, players can see not only their own resource levels but also those of the individuals with whom they have been paired, mimicking the situation among the Maasai in which livestock holdings are publicly visible. At the beginning of each round, players are all given the same amount of resources. During each period, the change in each player’s holdings is determined by how much the player harvests, how much the player received from the other player, how much the player gives to the other player, a natural growth rule, and a random shock. Shocks can be both positive and negative and average zero. Shock sizes are independent of the size of players’ resource stocks. The natural growth rule is a function of a rate parameter, the size of a player’s resource stock after harvest, and upper and lower critical values of the growth rule. During half the rounds, resource volatility was high, with a rate parameter equal to 0.25 and upper and lower critical values of the growth rule set at 25 and 8, respectively. During the other half of the rounds, resource volatility was low, with a rate parameter equal to 0.05 and upper and lower critical values of the growth rule set at 25 and 2, respectively (see [45] for complete details regarding the mathematics of the game). The order of high volatility and low volatility rounds was counterbalanced among participants and experimental conditions. Before each round, players were provided with a few tips regarding how best to manage their resources, all of which were identical across all players. In the low volatility rounds, players were informed that their resource stocks would grow fastest if they kept them around 17 units, that they would grow less quickly if they deviated from that number, and that they would shrink if they fell below 2 or grew greater than 25. In the high volatility rounds, players were informed that their resource stocks would grow fastest if they kept them around 18 units, that they would grow less quickly if they deviated from that number, and that they would shrink if they fell below 8 or grew greater than 25. Partners were reassigned after each round. During each period, players have opportunities to decide how much, if any, to harvest, whether to ask their anonymous partner to provide them with additional resources, and whether and how to respond to any requests received from their anonymous partner. Players earned $5 simply for showing up for the experiment. They earned one additional dollar for every fifteen units of the resource they harvested.

Priming and framing experimental games has been shown many times to have an impact on how people play them. For example, simply adding the sentence “Note that your recipient relies on you” (“*Recuerda él está en tus manos*”) to the instructions for a Dictator Game has been found to increase generosity [46]. Such impacts mean that priming and framing may also be good ways to create common knowledge. In the present study, we followed the example of earlier work on osotua using the Trust Game [22,23] and had participants read priming texts before playing the game. In the Trust Game experiments, participants in the control condition read a text unrelated to the game in case simply reading a text, regardless of its content, might have an impact on how they play the game. In the present experiment, we chose not to include such a dummy text because of the different nature of the two games. Because players in the Trust Game make only a single decision, it is possible that simply reading a text, regardless of its content, could influence their game play. In the Risk-Pooling Game, in contrast, players make many decisions (whether to harvest, whether to ask for help, whether to give help, etc.) in period after period and round after round of play, which in our view greatly reduces the likelihood that simply reading a text, regardless of its content, would influence their game play. Thus, participants in the control condition read no priming text and played games that were not labeled. Participants in the primed condition read one of two priming texts. One text described the Maasai and their osotua system. Because we were also interested in whether priming and framing effects might be influenced by cultural familiarity, we used an account of hay sharing among ranchers in Paradise Valley, Nevada [47] to design a text that was essentially the same as the Maasai text except that it concerned ranchers in the American West. Half of the participants in each of these primed conditions then played games that were not labeled. The other half played games that were labeled either as “The Osotua Game” or “The Rancher Game,” depending on which of the two texts they had read (see Table 2 for a summary of the experimental design). Priming and framing conditions, or the lack thereof, were the same for each pair of players. To ensure that players had an incentive to read the priming texts, they were also told that after the game they would have to take a multiple choice quiz regarding the content of the text they had read. Players were not given any additional incentive to do well on the quizzes. The mean score on all of the quizzes combined was 78.4% correct. The priming texts, instructions, and quizzes are all provided in the Supporting Information.

**Table 2:** Experimental design.

	Control	Experimental treatments			
Text read	None	Maasai	Maasai	Rancher	Rancher
Game label	None	None	The Osotua Game	None	The Rancher Game

In October and November, 2011, we recruited 198 players using an online recruiting system for the Wachtler Experimental Economics Laboratory at Rutgers University. Registrants in the system, all of whom were undergraduate students at Rutgers University-New Brunswick, in business, economics and other arts and sciences majors, were contacted via online announcements about the study, and interested subjects then signed up for specific sessions through the online system. There were no inclusion or exclusion restrictions except that subjects could participate in only one session. Players ranged in age from 18 to 37, with a mean age of 20.18 years. 125 players identified themselves as male and 73 as female. Forty players participated in each of the five experimental conditions with the exception of the rancher-framed condition, which had only thirty-eight. Because ANOVA tests with Scheffe post-hoc analyses revealed no significant differences among the experimental conditions, our results and analyses pool the experimental conditions into what we refer to as simply the primed condition. There is no necessary presumption that the sample of participants we recruited are representative of the population of, say, the United States because participants self-selected into the experiment and freely chose to participate, as is always the case for experiments of this sort performed using undergraduate students as participants. However, as we were careful to randomly assign participants to different treatments in the experiment, differences in behavior between treatments can be reliably considered to be due to the treatments and not due to selection bias into specific treatments.

## Results

To assess whether the players in the primed conditions followed a strategy more in line with need-based transfers than with debt-based transfers, we examined seven variables generated by the Risk Pooling Game:

1. *Repetitive Giving*: The number of periods in which the first player gave to the second player even though the second player had not yet repaid a previous gift.
2. *Repetitive Asking*: The number of periods in which the first player asked for help from the second player even though the first player had not yet repaid a previous gift.
3. *Mean Amount Requested*: The amount requested by a player averaged over all rounds and periods of game play.
4. *Mean Amount Given*: The amount given by a player averaged over all rounds and periods of game play.
5. *Number of Requests Made*: The number of times a player asked for a transfer of resources from the other player.
6. *Number of Requests Responded to Positively*: The number of times a player, when asked, did give something to the other player.
7. *Matching between Amounts Given and Received*: The differences between amounts given and received, in absolute values.

Regarding those variables, we predicted that the need-based transfer primed conditions would have the following effects compared to the control condition:

1. More *Repetitive Giving*
2. More *Repetitive Asking*
3. Higher *Mean Amounts Requested*
4. Higher *Mean Amounts Given*
5. Higher *Number of Requests Made*
6. Higher *Number of Requests Responded to Positively*
7. Less *Matching between Amounts Given and Received*

While players in the control condition might be reluctant to give to the other before previous transfers have been repaid or might be reluctant to request again before they have repaid previous transfers, players in the primed conditions might have a better understanding that keeping track of debts and repayments is inappropriate when the future is uncertain and that it is more important simply to keep the other player alive. This same logic may lead to a willingness to request more, to give more, to make more requests, to respond more positively to requests received, and to pay less attention to whether transfers between players are equal over time. In addition, because our computer simulations have indicated that agents who use a need-based transfer strategy survive longer than players using a debt-based strategy, we examined the number of periods each player survived during the game.

**Fig 1:**
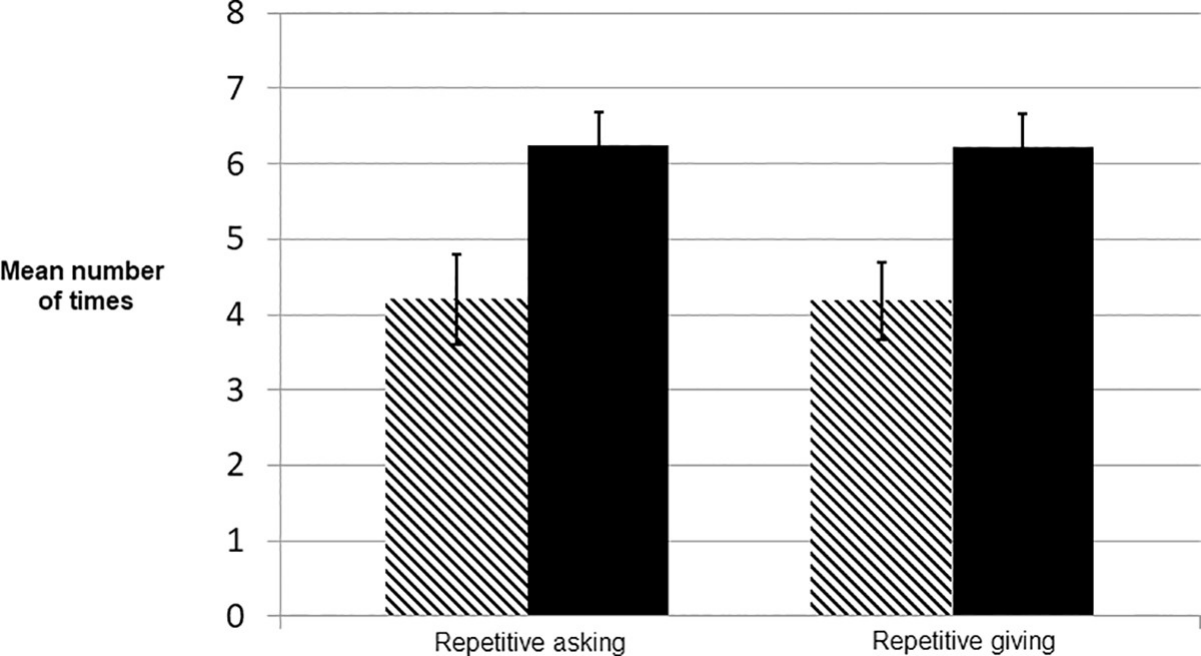
Repetitive asking and giving. Mean numbers of times players asked for resources without first repaying for resources they had already received (repetitive asking; t = -2.759, p = 0.007) and gave resources without first having been repaid for resources they had already given (repetitive giving; t = -3.035, p = 0.003). Solid bars = primed conditions, hashed bars = control condition. Error bars represent the standard errors of the means.

**Fig 2:**
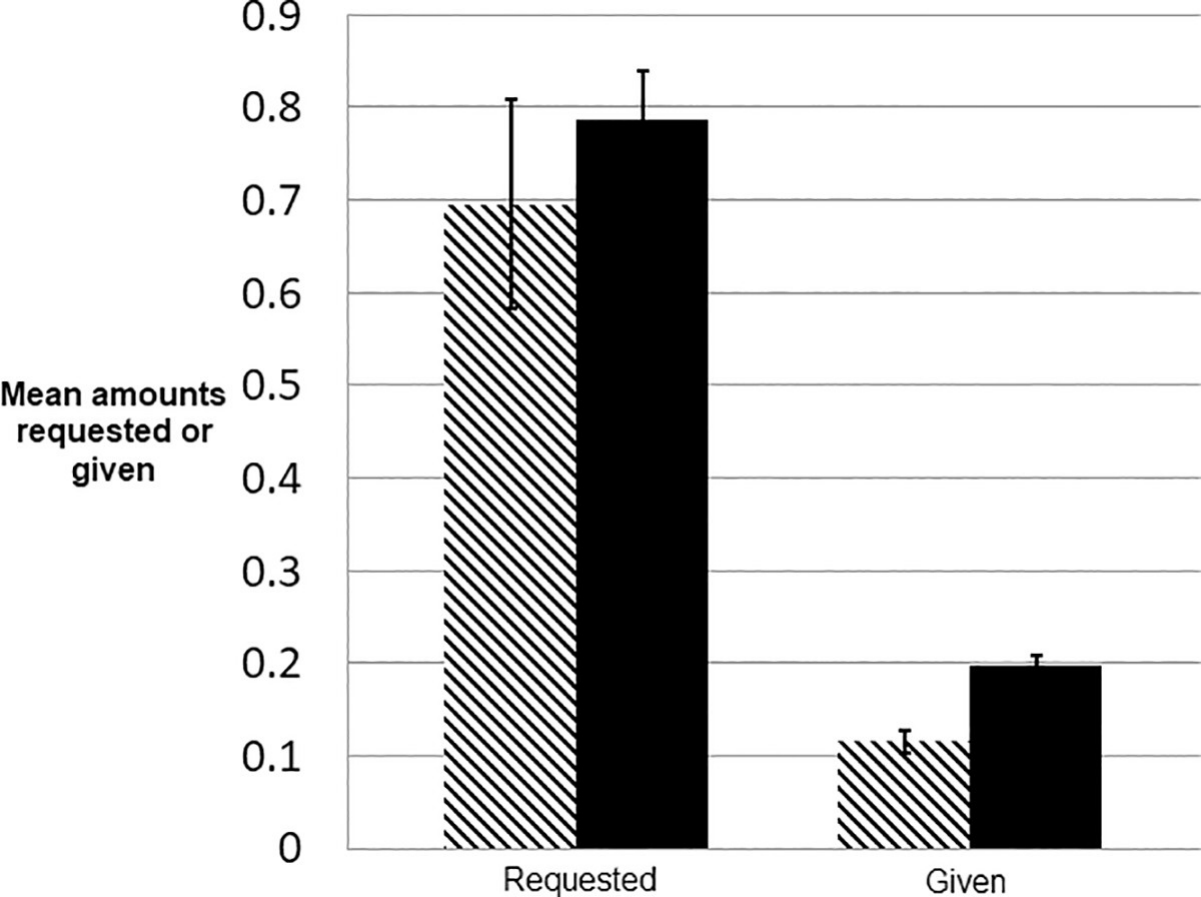
Amounts requested and given. Amounts requested (t = - 0.765; p (two-tailed) = 0.445) and given (t = -3.314, p (two-tailed) < 0.001) averaged over all periods. Solid bars = primed conditions, hashed bars = control condition.

**Fig 3:**
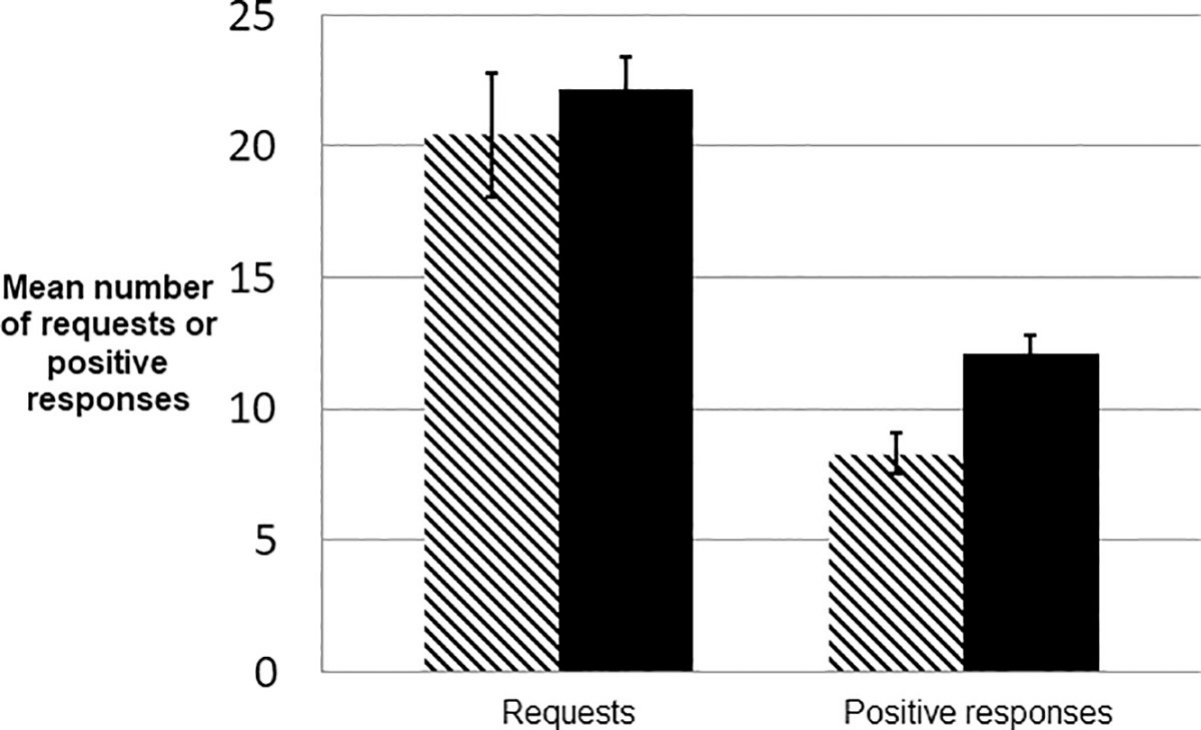
Requests and positive responses. Players in the primed condition (solid bars) made more requests and more often responded positively to requests than those in the control (hashed bars) condition. The difference in the number of times players made requests is not significant (t = -0.86; p (two-tailed) = 0.3936), but the difference in the number of times such requests were responded to positively was significant (t = -3.944; p (two-tailed) = 0.000), suggesting (as in Fig 2) that the priming texts may have had a larger effect on giving behavior than asking behavior. Error bars represent the standard errors of the means.

**Fig 4:**
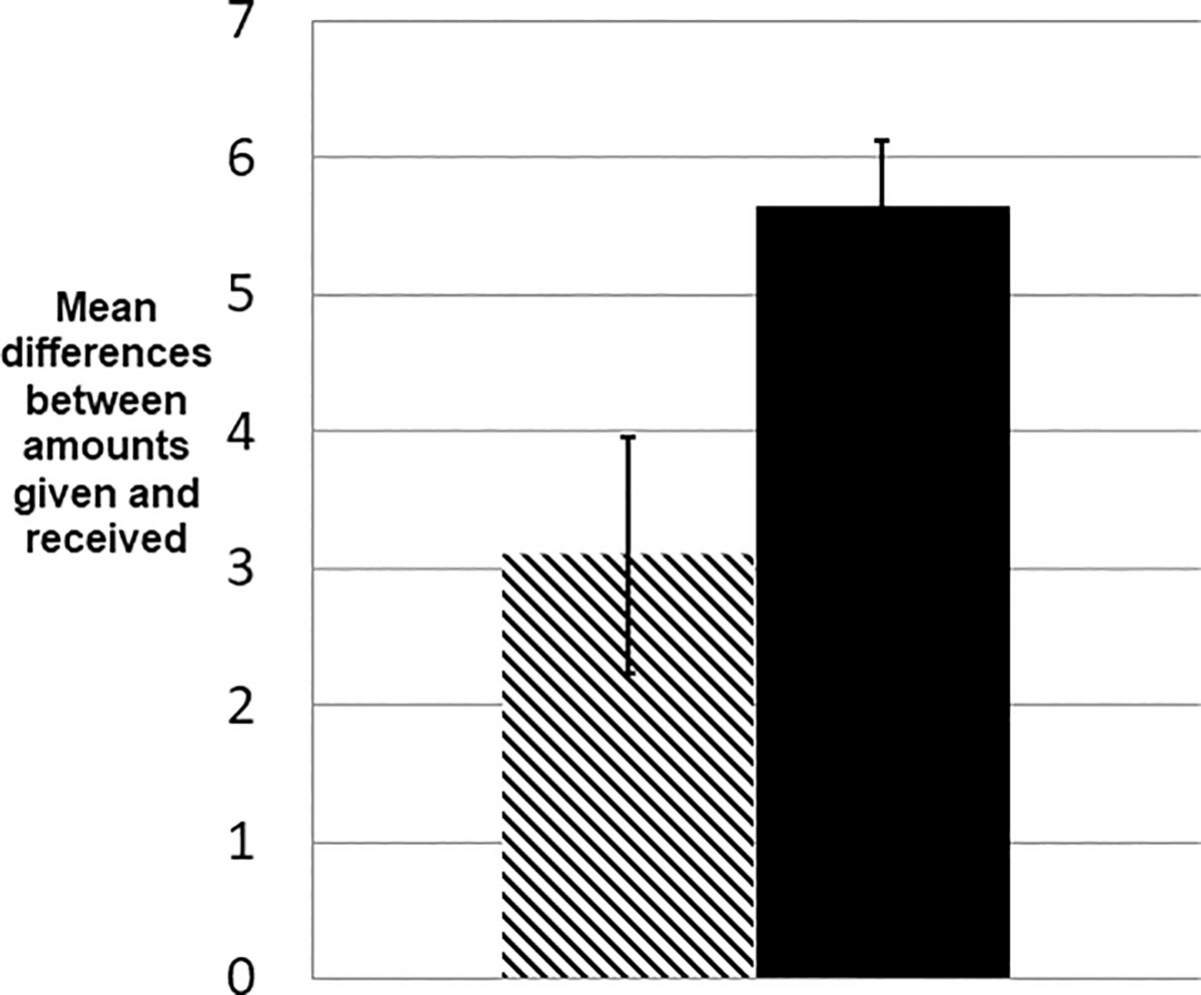
Differences between amounts given and received. Players in the primed condition (solid bar) had greater differences between the amounts they gave and the amounts they received, possibly reflecting greater tolerance for unbalanced accounts than in the control condition (hashed bar). The absolute differences between the amounts players gave and received over all rounds is significantly higher in the primed condition (t = -2.594, p (two-tailed) = 0.012). Error bars represent the standard errors of the means.

We used SPSS version 23 and the Simple Interactive Statistical Analysis web site [48] to analyze the data as a series of independent samples t-tests for differences between means. The results are presented in Figs 1–4.

Fig 1 shows that the priming texts reduced the extent to which players kept accounts, repaid debt, and engaged in tit-for-tat play. Players in the primed condition, on average, were more likely to ask for resources even if they had not yet repaid their partners for resources that they had already been given. They were also more likely to give to a partner in need even if they had not yet been repaid for resources they had already given. This suggests that the priming texts did indeed enhance need-based transfer behavior, perhaps by helping players to understand the nature of the problem they faced and the value to themselves in the long run of keeping the other player in the game.

As shown in Fig 2, players in the primed condition request more and gave more to their partners. The difference between mean amounts given was significant (p < 0.001), though the difference between amounts requested was not significant (p = 0.445). This suggests that the priming texts may have a large effect on willingness to give, but less of an effect on willingness to ask. Error bars represent the standard errors of the means.

Players in the primed condition made more requests and responded more to their partners (Fig 3). Although the difference in requests is not statistically significant (p = 0.3936), the difference in positive response rate is (p = 0.001).

As shown in Fig 4, the priming text leads to greater differences between the amounts players gave and received. This suggests again that players in the primed condition may be more tolerant of unbalanced accounts.

Contrary to our predictions, we did not discover a difference between the players in the control and primed conditions in terms of how many periods they survived. In both conditions, more than half of the players survived all eighty periods, leading to a ceiling effect that may have compromised our ability to detect any impact the priming texts may have had on survival. The ease with which players survived may also help explain the fact that the different degrees of volatility in different rounds had no statistically significant impact on, for example, the amounts players gave one another [45].

Because we are making multiple comparisons, it is appropriate also to adjust our alpha level, i.e., the critical p-value at which we claim statistical significance. Bonferroni’s [49] correction for multiple comparisons entails dividing our current alpha (0.05) by the number of comparisons we are making (seven), which yields an adjusted alpha of 0.007. The only impact this has on our claims of statistical significance is for the finding that the priming texts led to less matching between amounts given and received (p = 0.012). However, because the Bonferroni procedure reduces statistical power, some argue that it should be set aside and that effect size estimates should be presented, instead [50]. We do that in the next section (Table 3).

**Table 3:** Effect size estimates. Cohen’s d for our seven main variables as calculated by the Social Science Statistics (http://socscistatistics.com) web site.

Variable	Cohen’s d
Repetitive Giving	0.46
Repetitive Asking	0.44
Mean Amount Requested	0.13
Mean Amount Given	0.65
Number of Requests Made	0.13
Number of Requests Responded to Positively	0.60
Matching Between Amounts Given and Received	0.45

## Discussion

To explore the impact of common knowledge on social coordination and risk pooling in volatile environments, we designed and ran a Risk Pooling Game. We found that subjects who read texts about real-world risk pooling systems followed a strategy that is more in line with need-based transfers than did subjects in the control condition. Participants in the primed condition responded more positively to requests, gave more, engaged in more repetitive asking and giving, and tolerated greater imbalances between amounts given and received than those in the control condition. This suggests that the priming texts moved subjects away from account-keeping behavior, where a balance of debt and credit is expected, to need-based transfer behavior.

Although we found significant effects of the priming texts on many of our variables, there was no significant effect on the likelihood or size of requests that individuals made. In this risk pooling game, requests are generated primarily by players’ sense of need after having experienced sudden decreases in their resources. Since the frequency and severity of such shocks were the same across all conditions, it is possible that number of requests made and the amounts requested were driven mostly by the shocks in the game rather than by differences in players’ strategies attributable to the impact of the priming texts.

Table 3 presents estimates of the effect sizes for our seven main variables. For those variables whose mean differences achieve conventional levels of statistical significance, effect sizes are moderate, ranging from a low of 0.44 for Repetitive Asking to a high of 0.65 for Mean Amount Given. One possible interpretation of this pattern is that, while the impact of the priming texts is statistically discernable, most players in the control condition also seem to have been able to figure out that it is better for one’s own survival in the game to keep the other player in the game as well rather than being overly concerned with debt and repayment. Thus, even in the absence of a priming text about risk-pooling systems in the real world, a norm of risk-pooling through transfers to those in need may emerge as players familiarize themselves with the game and the volatility of the virtual environment in which it is played.

We have also seen this pattern in how people play a similar game set up as an interactive exhibit in San Francisco’s Exploratorium museum [51]. In the Survival Game exhibit, two to four museum visitors are provided with virtual livestock herds that grow and shrink stochastically from round to round, along with the opportunity to request cattle from other players and to respond to such requests. As in the Risk Pooling Game, players who fail to maintain their herds above a fixed threshold fall out of the game. Observations of people playing the game indicate that most visitors quickly grasp the logic of risk-pooling through transfers to those in need, i.e., that it is better for your own long term survival to be generous to those in need because it is quite likely that you yourself may be the needy player in the next round. Because the Survival Game is played on a computer interface, it is capable of recording the details of how visitors play the game, and we have plans to analyze those data for a future publication.

In most experimental studies of cooperation or sharing (e.g., in Prisoner’s Dilemma and Trust Games), matching of amounts given and received and other tit-for-tat-like behaviors are typically considered to be evidence of cooperation. However, as previous modeling work demonstrates, risk pooling can be better achieved through transfers to those in need without the creation of debt [24–26]. Thus, our conception of cooperation and how it is achieved should be expanded to accommodate this pattern of need-based transfers in addition to debt-based models of balanced reciprocity.

This leads to an obvious question: When should people engage in need-based transfers rather than debt-based transfers, and vice versa? Results from computer models and fieldwork suggest that, when needs arise regularly and predictably, it may be feasible and sensible to establish tit-for-tat relationships in which help given is expected to be repaid at some future time [6]. However, when needs arise unpredictably, as in our computer simulations and the Risk Pooling Game, it is sensible simply to help those in need if one is able to do so without putting one’s own welfare in jeopardy. Helping those in need fills the role of an insurance policy, substituting the certainty of a small, manageable loss in the present for the uncertainty of a large, catastrophic loss in the future.

An example of this contrast between debt-based and need-based transfers is provided by ethnographic fieldwork currently underway among ranchers in Cochise County, Arizona and Hidalgo County, New Mexico [52]. Ranches in this area typically cover many square miles but are managed by just a single family. Although most ranch families can provide enough skilled workers to deal with daily chores, few can provide the labor needed for such large chores as branding and shipping cattle. Although some do hire cowboys, many complain about the difficulty of finding people who still have the skills necessary to do the work efficiently and safely. However, one source of skilled labor is readily available: Neighboring ranches. Not only do neighbors have the skills, they also have the same need. This creates a perfect situation for a regular, steady exchange of labor. Ranchers refer to this as “trading out work” or “neighboring.” Neighbors, who may live as much as two hours’ drive apart from each other, negotiate with each other regarding the dates certain types of work will be done, and then they show up and help. In such situations, there are two unstated expectations: first, that they will be fed, and, second, that they will receive similar help when they need it on their ranch. This corresponds to a debt-based transfer system, where accounts are kept and individuals are expected to pay one another back in kind.

Unpredictable needs also often arise among the ranchers, most commonly due to the injuries they suffer due to the dangers inherent in the job. When such needs arise, neighboring ranchers come to the aid of the needy ranch with no questions asked and no expectation of any return apart from a similar generosity should they ever be in a similar bind. This kind of behavior is simply seen as being neighborly, and no accounts are kept or debts created. This corresponds to need-based transfer behavior.

Risk pooling arrangements such as those found among Maasai and American ranchers may provide ancillary benefits through the creation of social capital, defined as “relations of trust, reciprocity, common rules, norms and sanctions, and connectedness in institutions” [53]. Such social capital can subsequently aid in the creation of community resource management schemes and other forms of collective action [53,54]. For example, ranchers in southeastern Arizona and southwestern New Mexico have formed the Malpai Borderlands Group, a nonprofit organization that works with the Nature Conservancy, the US Forest Service, the Bureau of Land Management, and other stakeholders in the area to sustainably manage rangelands in their area [55]. Appropriately for this article, the Malpai group has even been the basis for educational exchanges between American ranchers and Maasai herders [56].

Through this fieldwork in the American West and other sites that are part of The Human Generosity Project, we are investigating the prevalence of need-based transfers, as well as the conditions under which need-based transfers vs. debt-based transfers occur in human societies. We are also continuing to investigate the viability of need-based and debt-based transfers under different ecological conditions using computer modeling. Our laboratory work continues as well, as we are now investigating how humans detect cheating in need-based transfers systems and how this cheater detection can help to stabilize cooperation [57]. In the future, we hope to use a game similar to the one described in this article to explore how people choose their risk-pooling partners. Such an experiment would involve, first, having players make decisions about how best to manage their resources that involve tradeoffs between risks and rewards, and, second, giving them the power to select their own risk-pooling partners. We would predict that players with similar risk preferences will tend to find each other and pair up. Such an experiment would also provide an opportunity to study hormonal profiles and other biological factors that may influence people’s willingness to take risks [58].

## Supporting information

S1 File Priming texts, instructions, tips, and quizzes. (DOCX)

S2 File Data in excel format. (XLSX)
