# Supplementary material for: Common knowledge promotes risk pooling in an experimental economic game
Source: PLoS One. 2019 Aug 15;14(8):e0220682. doi: 10.1371/journal.pone.0220682 (PMC6695222; doi:10.1371/journal.pone.0220682)
Supplement: S1 File — (DOCX) [file pone.0220682.s001.docx]

**Supporting information: Priming texts, instructions, tips, and quizzes**

Participants in the control condition read no priming text and played games that were not labeled. Participants in the primed condition read one of two priming texts. One text described the Maasai and their osotua system. Because we were also interested in whether priming and framing effects might be influenced by cultural familiarity, we used an account of hay sharing among ranchers in northern Nevada (Marshall and Ahlhorn 1980) to design a text that was essentially the same as the Maasai text except that it concerned ranchers in the American West. Half of the participants in each of these primed conditions then played games that were not labeled. The other half played games that were labeled either as “The Osotua Game” or “The Rancher Game,” depending on which of the two texts they had read. Priming and framing conditions, or the lack thereof, were the same for each pair of players. To ensure that players had read the priming texts, they also had to respond to a ten-question multiple choice quiz regarding the content of the text they had read before moving on to play the game.

1. Maasai Priming Text

Herding and Risk among the Maasai

This short essay will familiarize you with the basic features of herding among the Maasai. After reading it, you will play a game. After the game, you will take a short quiz to assess your retention.

The Maasai live in the East African countries of Kenya and Tanzania. They live mainly in and around the Great Rift Valley, which runs north and south through eastern Africa. The Great Rift Valley is a region dominated by vast grasslands. The Maasai make use of this landscape by basing their economy on livestock who can graze these grasslands. By definition, herders around the world make a living by raising livestock. Cattle are the most common type of livestock raised in herds, but many other species, including sheep, goats, camels, llamas, alpacas, and even reindeer, are also raised. Maasai herds typically consist mainly of cattle, goats, and sheep. Some herders make a living directly from the milk and meat of the animals they raise, while others sell their animals on the market. Some herders work on a very small scale, keeping just enough livestock to sustain a single family, while others own thousands of animals. But despite this diversity, the fact that they all raise livestock leads herders around the world to share a surprising number of common characteristics.

One thing that is true of almost all herders is that they live in marginal environments with climates unsuitable for raising crops. The most common places to find them are in arid and semi-arid regions such as the Great Rift Valley. Wherever land is not suitable for raising crops but still provides something for livestock to eat, you are likely to find herding. Because the land they live on is not very productive, Maasai herders usually live at low population densities, spread thinly across the landscape.

Often, herders are far from the protection of police forces or other representatives of government authority. For that reason, they are often willing and able to defend their livestock and other property on their own, without help from outsiders. This leads to a degree of in- dependence and freedom that many herders enjoy, despite the risks that accompany it. The dry regions in which most Maasai herders live tend to be prone to droughts. Unpredictable and sometimes severe losses of livestock from droughts, diseases, and theft can make herding a risky way to make a living. As a result, a person who is wealthy one year might be poor the next. Maasai herders have found a variety of ways to insulate themselves from the risks associated with these severe losses.

One way of reducing these risks is to participate in a system of resource transfer, typically of goods or livestock. This system is based on honor, respect and restraint. Individuals ask for help only if they are in genuine need (limiting their request to what is actually needed), and those who are asked for help are bound by honor to give if they can. There is no expectation that gifts will necessarily balance out over time. If one partner is unlucky and thus in need more often than the other one, then transfers may be mostly in one direction. As a result, gifts in this system do not create debt and are not seen as payments. These relationships are seen as great responsibilities, and the partners treat each other with great respect. Asking for something that you do not really need or refusing to give when you can afford to do so are both seen as violations of the rules of the system. Computer models suggest that this system helps each individual herder maintain a viable herd longer despite the problems of drought, disease, and theft because of the way that it insulates individuals from the variability of the environment.

This system of risk pooling is called *osotua*. The literal meaning of osotua is umbilical cord, so by using it to refer to gift-giving relationships Maasai are making a metaphorical connection between such relationships and the life-giving relationship between a mother and her child. Osotua relationships are some of the most important ties that Maasai have with each other, and osotua as a principle is one of the cornerstones of Maasai social life.

In the environments in which the Maasai live, there are often local droughts and diseases that affect some herders more severely than others. During any given year, one herder may find himself with a surplus of livestock while another may have too few livestock to support his family. In the osotua system, a herder in this predicament can ask a more fortunate herder for assistance. The more fortunate herder would then fulfill the request as long as he had enough livestock to fulfill the request without putting his own family in jeopardy, and would not expect repayment since osotua is not a system of debt.

1. Rancher Priming Text

Ranching and Risk in the American West

This short essay will familiarize you with the basic features of ranching in the American West. After reading it, you will play a game. After the game, you will take a short quiz to assess your retention.

American ranchers live in the arid and semi-arid regions of the western United States, including parts of Texas, Arizona, New Mexico, California, Nevada, Utah, Colorado, Wyoming, and Montana. They live mainly in regions dominated by grasslands. American ranchers make use of this landscape by basing their economy on livestock that can graze these grasslands. By definition ranchers around the world make a living by raising livestock. Cattle are the most common type of livestock raised on ranches, but many other species, including sheep, goats, camels, llamas, alpacas, and even reindeer, are also raised. American ranchers raise mostly cattle and sheep. Some ranchers make a living directly from the milk and meat of the animals they raise, while others sell their animals on the market. Some ranchers work on a very small scale, keeping just enough livestock to sustain a single family, while others own thousands of animals. But despite this diversity, the fact that they all raise livestock leads ranchers around the world to share a surprising number of common characteristics.

One thing that is true of almost all ranchers is that they live in marginal environments with climates unsuitable for raising crops. The most common places to find them are in arid and semi-arid regions such as the American West. Wherever land is not suitable for raising crops but still provides something for livestock to eat, you are likely to find ranching. Because the land they live on is not very productive, American ranchers usually live at low population densities, spread thinly across the landscape.

Often, ranchers are far from the protection of police forces or other representatives of government authority. For that reason, they are often willing and able to defend their livestock and other property on their own, without help from outsiders. This leads to a degree of independence and freedom that many ranchers enjoy, despite the risks that accompany it. The dry regions in which most American ranchers live tend to be prone to droughts. Unpredictable and sometimes severe losses of livestock from droughts, diseases, and theft can make ranching a risky way to make a living. As a result, a person who is wealthy one year might be poor the next. American ranchers have found a variety of ways to insulate themselves from the risks associated with these severe losses.

One way of reducing these risks is to participate in a system of resource transfer, typically of goods or livestock. This system is based on honor, respect and restraint. Individuals ask for help only if they are in genuine need (limiting their request to what is actually needed), and those who are asked for help are bound by honor to give if they can. There is no expectation that gifts will necessarily balance out over time. If one partner is unlucky and thus in need more often than the other one, then transfers may be mostly in one direction. As a result, gifts in this system do not create debt and are not seen as payments. These relationships are seen as great responsibilities, and the partners treat each other with great respect. Asking for something that you do not really need or refusing to give when you can afford to do so are both seen as violations of the rules of the system. Computer models suggest that this system helps each individual rancher maintain a viable herd longer despite the problems of drought, disease, and theft because of the way that it insulates individuals from the variability of the environment.

Ranchers in the American West reduce their exposure to risk by pooling it with fellow ranchers. Risk-pooling serves basic and central values of ranchers as it is based on honor and respect. It also promotes their autonomy by improving the robustness of their capacity to respond to the uncertainty in the environment without intervention from centralized government.

One good example of this risk-pooling system comes from a study of ranchers and their buckaroos, also known as cowboys, in northern Nevada. During the summer, ranchers there grow hay to feed their livestock during the regions severe winters. Local variation in drought and disease can lead to high variability in the amount of hay a given rancher can grow in a given summer. During any given year, one rancher may find himself with a surplus of hay while another may be short. In the risk-pooling system, a rancher in this predicament can ask the more fortunate rancher for assistance. The more fortunate rancher would then fulfill the request, as long as the existing surplus of hay was great enough to give without putting his herd in jeopardy. The rancher giving this hay would not expect repayment since risk-pooling is not a system of debt.

1. Instructions for the unlabeled games

Introduction

You are about to participate in an experiment to study how people make decisions when faced with uncertainty. Various research foundations have provided the funding for this study. You will be paid for your participation and according to your performance in the game, as explained below.

Playing the Game

Your goal is to manage and harvest a resource over time. You will play four separate rounds, in each of which there will be 20 decision making periods. During each period, the resource level will change based on the growth of the resource. If the resource level is too low, it will shrink rather than grow. Likewise, if the resource level is too high, it will shrink rather than grow. By harvesting the right amount of the resource, you can keep it at a level where it will grow quickly. However, there are also random changes to your resource level, called Shocks. These shocks may make it harder to manage your resource by unexpectedly increasing or decreasing the size of your stock. Each time period you will have the opportunity to ask for resources from another participant, and another participant may request resources from you. Your earnings in each round will be based on the total amount of resources that you harvested over all the time periods in that round, and the total size of your current stock at the end of the round.

After an initial practice round of 7 periods in which there are no random shocks and no ability to request, we will begin the first paying round. Each period will be made up of several separate steps/decisions:

1. Make Harvest Decision: You will be asked to decide how much of your resource you want to harvest in the current time period.
2. Option to Make Request: You will have an opportunity to make a request from the person with whom you are paired.
3. Option to Fulfill Request: You will have the opportunity to transfer resources to your partner if your partner made a request.
4. Natural Growth Rule Applied to Resource stock: Once all decisions are made, the natural growth of your resource stock will be calculated.
5. Random Shock Applied: The random shock to your resource level will be determined and applied to your resources.
6. Feedback: The total of your natural growth plus your random shock will be displayed, along with the resulting final level of your resource stock for the period. You will also be able to see this same information about the person you are paired with, and vice-versa.\
7. System Checks Resource Level: If your resource level is 0 for two consecutive time periods, your play of the current round will end.

Understanding Random Shocks

Because of shocks, your resource might increase (positive shock) or decrease (negative shock) unexpectedly. The average size of these shocks is 0, but since there are both positive and negative shocks, there will often be rounds when your resource level changes in an unpredictable way. The size of the random shock is independent of size of your resource stock. However, if you have a large negative shock, this may reduce your resource stock to 0. These random shocks will require you to think more carefully about how much you harvest during a given time period. If you have a large loss, you might want to harvest less and if you have a large gain, you might want to harvest more.

Requests for Resources

You will be paired with another participant at the start of each round (each round is a set of 20 time periods). You will manage your own resource stock, and the participant with whom you are paired will manage his/her own resource stock. However, you will have the opportunity during every time period to make a request for some amount of resources from the other participant, and the other participant can also make a request from you. If a request is made of you, you will have the opportunity to respond to that request by giving the other participant an amount of resources, which you can specify (it can be the same as, less than, or more than the request amount). The other participant will also have the opportunity to fulfill your request in the same fashion.

Your earnings for the Game

You will earn $5 for having shown up to the experiment today. For every 15 units of the resource that you harvest, you will earn one U.S. Dollar. For example, if you manage to harvest an average of 60 resource units per 20 period round (including the leftover capital stock), then you would earn (4 x 60) = 240 game points, which is $16 US, plus the $5 show up payment for a total of $21 US. Earnings may vary due to random luck and the exact decisions that you make, so this is not a guarantee, just an estimate.

(4) Instructions for the labeled games

The Osotua [or Rancher] Game

You are about to participate in an experiment to study how people make decisions when faced with uncertainty. Various research foundations have provided the funding for this study. You will be paid for your participation and according to your performance in the game, as explained below.

Playing the Osotua [or Rancher] Game

Your goal is to manage and harvest a resource over time. You will play four separate rounds, in each of which there will be 20 decision making periods. During each period, the resource level will change based on the growth of the resource. If the resource level is too low, it will shrink rather than grow. Likewise, if the resource level is too high, it will shrink rather than grow. By harvesting the right amount of the resource, you can keep it at a level where it will grow quickly. However, there are also random changes to your resource level, called Shocks. These shocks may make it harder to manage your resource by unexpectedly increasing or decreasing the size of your stock. Each time period you will have the opportunity to ask for resources from another participant, and another participant may request resources from you. Your earnings in each round will be based on the total amount of resources that you harvested over all the time periods in that round, and the total size of your current stock at the end of the round.

After an initial practice round of 7 periods in which there are no random shocks and no ability to request, we will begin the first paying round. Each period will be made up of several separate steps/decisions:

1. Make Harvest Decision: You will be asked to decide how much of your resource you want to harvest in the current time period.
2. Option to Make Request: You will have an opportunity to make a request from the person with whom you are paired.
3. Option to Fulfill Request: You will have the opportunity to transfer resources to your partner if your partner made a request.
4. Natural Growth Rule Applied to Resource stock: Once all decisions are made, the natural growth of your resource stock will be calculated.
5. Random Shock Applied: The random shock to your resource level will be determined and applied to your resources.
6. Feedback: The total of your natural growth plus your random shock will be displayed, along with the resulting final level of your resource stock for the period. You will also be able to see this same information about the person you are paired with, and vice-versa.\
7. System Checks Resource Level: If your resource level is 0 for two consecutive time periods, your play of the current round will end.

Understanding Random Shocks

Because of shocks, your resource might increase (positive shock) or decrease (negative shock) unexpectedly. The average size of these shocks is 0, but since there are both positive and negative shocks, there will often be rounds when your resource level changes in an unpredictable way. The size of the random shock is independent of size of your resource stock. However, if you have a large negative shock, this may reduce your resource stock to 0. These random shocks will require you to think more carefully about how much you harvest during a given time period. If you have a large loss, you might want to harvest less and if you have a large gain, you might want to harvest more.

Requests for Resources

You will be paired with another participant at the start of each round (each round is a set of 20 time periods). You will manage your own resource stock, and the participant with whom you are paired will manage his/her own resource stock. However, you will have the opportunity during every time period to make a request for some amount of resources from the other participant, and the other participant can also make a request from you. If a request is made of you, you will have the opportunity to respond to that request by giving the other participant an amount of resources, which you can specify (it can be the same as, less than, or more than the request amount). The other participant will also have the opportunity to fulfill your request in the same fashion.

Your earnings for the Game

You will earn $5 for having shown up to the experiment today. For every 15 units of the resource that you harvest, you will earn one U.S. Dollar. For example, if you manage to harvest an average of 60 resource units per 20 period round (including the leftover capital stock), then you would earn (4 x 60) = 240 game points, which is $16 US, plus the $5 show up payment for a total of $21 US. Earnings may vary due to random luck and the exact decisions that you make, so this is not a guarantee, just an estimate.

1. Tips provided to players moving from low risk games to high risk games

Practice Round Tips

Tips for managing your resource stock

In order to maximize your earnings in the game, you should maintain your resource at the level at which it grows most quickly. Your resource grows most quickly if you harvest enough to keep it around 17. If your resource is higher or lower than 17, it will grow less quickly. If your resource falls below 2, it will shrink in subsequent time periods. If your resource level is higher than 25, it will shrink as well. Your earnings will be based both on the amount that you harvested and the remaining resource stock at the end of each round, so you do not lose money by refraining from harvesting all of the resource stock. Use this practice round to familiarize yourself with the resource behavior. This round will not count towards your payment.

Round 1 Tips

Start Paying Periods

You are now playing for money. Random shocks will be applied, and you will have the ability to request resources from your randomly matched partner.

Tips for managing your resource stock

In order to maximize your earnings in the game, you should maintain your resource at the level at which it grows most quickly. Your resource grows most quickly if you harvest enough to keep it around 17. If your resource is higher or lower than 17, it will grow less quickly. If your resource falls below 2, it will shrink in subsequent time periods. If your resource level is higher than 25, it will shrink as well. Your earnings will be based both on the amount that you harvested and the remaining resource stock at the end of each round, so you do not lose money by refraining from harvesting all of the resource stock.

Round 2 Tips

Tips for managing your resource stock

In this second round, you have been matched with a different partner. Again, in order to maximize your earnings in the game, you should maintain your resource at the level at which it grows most quickly. Your resource grows most quickly if you harvest enough to keep it around 17. If your resource is higher or lower than 17, it will grow less quickly. If your resource falls below 2, it will shrink in subsequent time periods. If your resource level is higher than 25, it will shrink as well. Your earnings will be based both on the amount that you harvested and the remaining resource stock at the end of each round, so you do not lose money by refraining from harvesting all of the resource stock.

Round 3 Tips

Tips for managing your resource stock

In this third round, you have again been matched with a different partner. However, the resource behavior has changed. In order to maximize your earnings in the game, you should maintain your resource at the level at which it grows most quickly. Your resource grows most quickly if you harvest enough to keep it around 18 (this is different than prior rounds). If your resource is higher or lower than 18, it will grow less quickly. If your resource falls below 8 (this is different from prior rounds), it will shrink in subsequent time periods. If your resource level is higher than 25, it will shrink as well. Your earnings will be based both on the amount that you harvested and the remaining resource stock at the end of each round, so you do not lose money by refraining from harvesting all of the resource stock.

Round 4 Tips

Tips for managing your resource stock

In this fourth round, you have again been matched with a different partner. The resource behavior is the same as the last round you played. In order to maximize your earnings in the game, you should maintain your resource at the level at which it grows most quickly. Your resource grows most quickly if you harvest enough to keep it around 18. If your resource is higher or lower than 18, it will grow less quickly. If your resource falls below 8, it will shrink in subsequent time periods. If your resource level is higher than 25, it will shrink as well. Your earnings will be based both on the amount that you harvested and the remaining resource stock at the end of each round, so you do not lose money by refraining from harvesting all of the resource stock.

1. Tips provided to players moving from high risk games to low risk games

Practice Round Tips

Tips for managing your resource stock

In order to maximize your earnings in the game, you should maintain your resource at the level at which it grows most quickly. Your resource grows most quickly if you harvest enough to keep it around 18. If your resource is higher or lower than 18, it will grow less quickly. If your resource falls below 8, it will shrink in subsequent time periods. If your resource level is higher than 25, it will shrink as well. Your earnings will be based both on the amount that you harvested and the remaining resource stock at the end of each round, so you do not lose money by refraining from harvesting all of the resource stock. Use this practice round to familiarize yourself with the resource behavior. This round will not count towards your payment.

Round 1 Tips

Start Paying Periods

You are now playing for money. Random shocks will be applied, and you will have the ability to request resources from your randomly matched partner.

Tips for managing your resource stock

In order to maximize your earnings in the game, you should maintain your resource at the level at which it grows most quickly. Your resource grows most quickly if you harvest enough to keep it around 18. If your resource is higher or lower than 18, it will grow less quickly. If your resource falls below 8, it will shrink in subsequent time periods. If your resource level is higher than 25, it will shrink as well. Your earnings will be based both on the amount that you harvested and the remaining resource stock at the end of each round, so you do not lose money by refraining from harvesting all of the resource stock.

Round 2 Tips

Tips for managing your resource stock

In this second round, you have been matched with a different partner. Again, in order to maximize your earnings in the game, you should maintain your resource at the level at which it grows most quickly. Your resource grows most quickly if you harvest enough to keep it around 18. If your resource is higher or lower than 18, it will grow less quickly. If your resource falls below 8, it will shrink in subsequent time periods. If your resource level is higher than 25, it will shrink as well. Your earnings will be based both on the amount that you harvested and the remaining resource stock at the end of each round, so you do not lose money by refraining from harvesting all of the resource stock.

Round 3 Tips

Tips for managing your resource stock

In this third round, you have again been matched with a different partner. However, the resource behavior has changed. In order to maximize your earnings in the game, you should maintain your resource at the level at which it grows most quickly. Your resource grows most quickly if you harvest enough to keep it around 17 (this is different than prior rounds). If your resource is higher or lower than 17, it will grow less quickly. If your resource falls below 2 (this is different from prior rounds), it will shrink in subsequent time periods. If your resource level is higher than 25, it will shrink as well. Your earnings will be based both on the amount that you harvested and the remaining resource stock at the end of each round, so you do not lose money by refraining from harvesting all of the resource stock.

Round 4 Tips

Tips for managing your resource stock

In this fourth round, you have again been matched with a different partner. The resource behavior is the same as the last round you played. In order to maximize your earnings in the game, you should maintain your resource at the level at which it grows most quickly. Your resource grows most quickly if you harvest enough to keep it around 17. If your resource is higher or lower than 17, it will grow less quickly. If your resource falls below 2, it will shrink in subsequent time periods. If your resource level is higher than 25, it will shrink as well. Your earnings will be based both on the amount that you harvested and the remaining resource stock at the end of each round, so you do not lose money by refraining from harvesting all of the resource stock.

1. Maasai quiz

The following are the quiz questions asked of participants who read the Maasai priming text. Correct answers are in bold.

1. The Maasai live in
2. Kenya.
3. Tanzania.
4. The Great Rift Valley.
5. **All of the above.**
6. The Maasai live in a region dominated by
7. swamps.
8. forests.
9. lakes.
10. **grasslands.**
11. The species of livestock raised by herders around the world include
12. cattle.
13. sheep.
14. llamas.
15. **all of the above.**
16. Maasai herds consist mainly of
17. **cattle, goats, and sheep.**
18. cattle and sheep.
19. camels.
20. llamas and alpacas.
21. Population densities among herders are usually
22. **low.**
23. moderate.
24. high.
25. all of the above, depending upon local circumstances.
26. The areas in which Maasai live tend to be prone to
27. floods.
28. **droughts.**
29. earthquakes.
30. all of the above.
31. In the Maasai risk-pooling system, requests for help
32. **are limited to what one actually needs.**
33. are usually made when no help is actually needed.
34. may be made whether or not help is needed.
35. none of the above.
36. In the Maasai risk-pooling system, a person who receives a request
37. **is expected to honor it, if possible.**
38. is expected to ignore it until it is repeated.
39. keeps track of the debt that results from the gift.
40. none of the above.

9) In the Maasai risk-pooling system, gifts between two herders

1. are expected to balance out over time.
2. **are not expected to balance out over time.**
3. create a patron-client hierarchy.
4. none of the above.

10) In the Maasai risk-pooling system, herders who receive gifts are expected to

1. repay them as soon as they can.
2. repay them, plus interest.
3. **honor future requests from their partners.**
4. none of the above.
5. Rancher quiz

The following are the quiz questions asked participants who read the Rancher priming text. Correct answers are in bold.

1. American ranchers live in
   1. Texas.
   2. Arizona.
   3. Colorado.
   4. **all of the above.**
2. American ranchers live in a region dominated by
   1. swamps.
   2. forests.
   3. lakes.
   4. **grasslands.**
3. The species of livestock raised by herders around the world include
   1. cattle.
   2. sheep.
   3. llamas.
   4. **all of the above.**
4. American ranchers’ herds consist mainly of
   1. cattle, goats, and sheep.
   2. **cattle and sheep.**
   3. camels.
   4. llamas and alpacas.
5. Population densities among herders are usually
   1. **low.**
   2. moderate.
   3. high.
   4. all of the above, depending upon local circumstances.
6. The areas in which American herders live tend to be prone to
   1. floods.
   2. **droughts.**
   3. earthquakes.
   4. all of the above.
7. In the risk-pooling system used by American ranchers, requests for help
   1. **are limited to what one actually needs.**
   2. are usually made when no help is actually needed.
   3. may be made whether or not help is needed.
   4. none of the above.
8. In the risk-pooling system used by American ranchers, a person who receives a request
   1. **is expected to honor it, if possible.**
   2. is expected to ignore it until it is repeated.
   3. keeps track of the debt that results from the gift.
   4. none of the above.
9. In the risk-pooling system used by American ranchers, gifts between two herders
   1. are expected to balance out over time.
   2. **are not expected to balance out over time.**
   3. create a patron-client hierarchy.
   4. none of the above.
10. In the risk-pooling system used by American ranchers, herders who receive gifts are expected to
    1. repay them as soon as they can.
    2. repay them, plus interest.
    3. **honor future requests from their partners.**
    4. none of the above.
